# Impairment of Autophagic Flux Participates in the Antitumor Effects of TAT-Cx43_266-283_ in Glioblastoma Stem Cells

**DOI:** 10.3390/cancers13174262

**Published:** 2021-08-24

**Authors:** Sara G. Pelaz, Claudia Ollauri-Ibáñez, Concepción Lillo, Arantxa Tabernero

**Affiliations:** 1 Instituto de Neurociencias de Castilla y León (INCYL), Universidad de Salamanca, Calle Pintor Fernando Gallego 1, 37007 Salamanca, Spain; saragutierrezpelaz@usal.es (S.G.P.); collauri@usal.es (C.O.-I.); conlillo@usal.es (C.L.); 2 Departamento de Bioquímica y Biología Molecular, Universidad de Salamanca, Campus Miguel de Unamuno, 37007 Salamanca, Spain; 3 Instituto de Investigación Biomédica de Salamanca (IBSAL), Hospital Virgen de la Vega, 10ª Planta, Paseo de San Vicente 58-182, 37007 Salamanca, Spain; 4 Departamento de Biología Celular y Patología, Universidad de Salamanca, Campus Miguel de Unamuno, 37007 Salamanca, Spain

**Keywords:** glioblastoma, autophagy, connexin43, c-Src, glioblastoma stem cells, cell-penetrating peptide

## Abstract

**Simple Summary:**

Autophagy is a process in which the cell recycles components that are not needed at that moment and uses the resulting elements to satisfy more urgent needs. Depending on the specific context, this can be beneficial or detrimental for tumor development. We found that in glioblastoma, the most lethal brain tumor, autophagy is upregulated and contributes to glioblastoma stem cell survival under starvation. Importantly, the antitumor peptide TAT-Cx43_266-283_ blocks autophagy flux, contributing to the death of glioblastoma stem cells. This peptide induces glioblastoma stem cell death in nutrient-deprived and complete environments, while the effect of other unsuccessful drugs for glioblastoma depends on nutrient context, supporting the potential of TAT-Cx43_266-283_ as a treatment to improve the lives of glioblastoma patients.

**Abstract:**

Autophagy is a physiological process by which various damaged or non-essential cytosolic components are recycled, contributing to cell survival under stress conditions. In cancer, autophagy can have antitumor or protumor effects depending on the developmental stage. Here, we use Western blotting, immunochemistry, and transmission electron microscopy to demonstrate that the antitumor peptide TAT-Cx43_266-283_, a c-Src inhibitor, blocks autophagic flux in glioblastoma stem cells (GSCs) under basal and nutrient-deprived conditions. Upon nutrient deprivation, GSCs acquired a dormant-like phenotype that was disrupted by inhibition of autophagy with TAT-Cx43_266-283_ or chloroquine (a classic autophagy inhibitor), leading to GSC death. Remarkably, dasatinib, a clinically available c-Src inhibitor, could not replicate TAT-Cx43_266-283_ effect on dormant GSCs, revealing for the first time the possible involvement of pathways other than c-Src in TAT-Cx43_266-283_ effect. TAT-Cx43_266-283_ exerts an antitumor effect both in nutrient-complete and nutrient-deprived environments, which constitutes an advantage over chloroquine and dasatinib, whose effects depend on nutrient environment. Finally, our analysis of the levels of autophagy-related proteins in healthy and glioma donors suggests that autophagy is upregulated in glioblastoma, further supporting the interest in inhibiting this process in the most aggressive brain tumor and the potential use of TAT-Cx43_266-283_ as a therapy for this type of cancer.

## 1. Introduction

Glioblastomas (GBMs) are the most lethal type of brain tumor, with a median survival of just 16 months [1,2]. GBMs progress rapidly and aggressively and exploit their complex microenvironment to sustain their highly infiltrative nature. A subset of cells, termed glioblastoma stem cells (GSCs), has been characterized as highly tumorigenic and therapy-resistant and are, therefore, considered responsible for tumor recurrence and, ultimately, GBM lethality [3,4,5].

Connexin43 (Cx43) is a ubiquitous integral membrane protein that forms gap junction channels and hemichannels, with a long cytoplasmatic C-terminal that exhibits a large interactome [6]. One of the proteins that interact with the C-terminal of Cx43 is c-Src, a non-receptor protein kinase that was the first proto-oncogene discovered [7] and is known to play an important role in the transforming phenotype of malignant glioma [8]. In glioma cells, the C-terminal of Cx43 recruits c-Src together with its physiological inhibitors CSK and PTEN, causing the inhibition of c-Src and its pleiotropic oncogenic pathways by decreasing the phosphorylation levels of Y416 (activating) and increasing those of Y527 (inhibitory) residues [9,10,11]. Importantly, GBM cells, specially GSCs, have low Cx43 expression and high c-Src activity [10,12,13,14,15]. Indeed, ectopic expression of Cx43 in glioma cells reduces their rate of proliferation [16], stemness [10,17], and tumor formation in vivo [18], and therefore Cx43 is considered a tumor suppressor protein.

To capitalize on the therapeutic potential of Cx43 inhibition of c-Src activity, we designed a cell-penetrating peptide containing the TAT sequence, for efficient cellular internalization [19], fused to residues 266-283 of Cx43, which encompass the SH3 binding domain of Cx43 (residues 274-283), responsible for binding to c-Src [20]. This cell-penetrating peptide, named TAT-Cx43_266-283_, inhibits c-Src activity in the same manner that Cx43 does and recapitulates the antitumor effects of c-Src inhibition by Cx43 [9,10]. On the contrary, another Cx43-based cell-penetrating peptide containing the SH3 binding motif and an identical number of residues to TAT-Cx43_266-283_ but starting at the 274 residue (TAT-Cx43_274-291_) does not recruit c-Src inhibitors and does not display antitumor activity, highlighting the specificity of the c-Src inhibition exerted by the TAT-Cx43_266-283_ peptide [9,14,21]. We have previously shown that TAT-Cx43_266-283_, by inhibiting c-Src, reverses the stem cell phenotype [10], impairs migration, invasion [14], and metabolic plasticity [21] of GSCs, without affecting healthy brain cells, and improves survival of GBM mice models [22].

Autophagy is an evolutionarily conserved catabolic process and an important response to stress conditions, such as starvation. Activation of autophagy leads to the clearance of various cytosolic components, including non-essential proteins and damaged organelles, which are degraded and recycled, thereby contributing to cell survival under nutrient-deprived conditions. There are several types of autophagy, including chaperone-mediated autophagy and microautophagy, in which only lysosomes are involved, and macroautophagy (hereby referred to as autophagy), which involves autophagosomes and lysosomes [23]. The autophagic pathway consists of several stages. First, upon autophagy induction, a phagophore, a double membrane that encloses and isolates cytosolic components, is nucleated following activation of Beclin-1 in complex with other regulatory proteins, such as AMBRA. Then, multiple ATG proteins (autophagy-related proteins) catalyze the formation of phosphatidyl-ethanolamine (PE)-conjugated microtubule-associated protein light chain 3 (LC3-II, phagophore-membrane bound) from LC3-I (non-conjugated, cytosolic), which serves as a docking site for autophagic cargo and adaptor proteins, such as p62 (also referred to as sequestosome-1, SQSTM1) [24]. Finally, the nascent autophagosome is closed and transported to fuse with lysosomes, acidic organelles that contain cathepsins and other proteases, which leads to cargo degradation (including LC3 and p62) and recycling of nutrients and metabolites [25,26].

The role of autophagy in cancer is complex with context-dependent effects: in premalignant lesions, enhancing autophagy might prevent cancer, while, in advanced cancers, autophagy constitutes a survival mechanism, and consequently, the vast majority of studies are focused on inhibiting autophagy as a therapeutic strategy (reviewed in [27]). In glioma, several studies based on high-throughput patient mRNA data have found several different autophagy-related gene signatures that can predict glioma outcome [28,29,30], and protein-based analysis of autophagic flux in patient samples suggests that GBM as a higher autophagic flux than low-grade glioma, and that enhanced flux correlates with worse patient survival [31,32,33,34]. Moreover, autophagy is highly active in dormant cancer cells, cells that undergo reversible growth arrest and await favorable environmental cues to resume proliferation, from a wide variety of cancer types, including breast, ovary, gastrointestinal tract, pancreas, and bone cancers, their respective mice tumor or xenograft models, and patient-derived samples (reviewed in [35]), and has been shown as a crucial mechanism for the survival of disseminated dormant breast cancer cells [36]. In GBM, a dormant glioma GSC subpopulation is enriched at GBM tumor borders and can drive tumor relapse after chemotherapy [37]. Moreover, starvation can induce dormancy in GBM cells, which is accompanied by enhanced autophagy [38].

Chloroquine (CQ), and its derivative hydroxychloroquine (HCQ), are clinically available drugs that inhibit autophagy by decreasing autophagosome/lysosome fusion [39], yet they can also have autophagy-independent effects, such as lysosome vacuolization and Golgi and endo-lysosomal system disorganization, particularly after long-time exposure [39]. CQ and HCQ have been clinically evaluated in many types of cancer [27], and initial clinical trials in GBM using either drug in combination with temozolomide and radiation showed promising results [40,41]. However, follow-up trials have yielded inconclusive results, with reasons including dose-limiting toxicity [42], off-target effects, or the need for more insightful patient selection criteria [27,43]. Hence, although inhibition of autophagy in a clinical setting appears promising, a better understanding of drug and tumor biology and new autophagy inhibitors are needed to obtain the desired effect on clinical outcomes.

Recently, we found that nutrient deprivation promotes a GSC dormancy-like state that was reverted upon nutrient replenishment [21]. As previously mentioned, this mechanism could be critical for GSC survival under stressful conditions. Importantly, treatment with TAT-Cx43_266-283_ previous to nutrient deprivation abolished GSC dormancy and promoted cell death [21]. In this study, we aimed to investigate the relevance of autophagy in GSC dormancy promoted by nutrient deprivation. We found that autophagy is necessary for GSCs under nutrient deprivation and that the autophagic arrest induced by TAT-Cx43_266-283_ contributed to cell death in this context. Finally, analysis of protein expression from low- and high-grade glioma patients suggested a higher autophagic flux in GBM than low-grade glioma, arguing in favor of autophagy blockage as a therapeutic strategy.

## 2. Materials and Methods

### 2.1. Glioblastoma Stem Cells

G166 human GSCs (RRID: CVCL_DG66) and G179 human GSCs (RRID: CVCL_DG69) were obtained from BioRep [10]. Unless otherwise stated, the GSCs used in the experiments were G166 GSCs. GSCs were in DMEM F-12 HAM (Sigma-Aldrich, St Louis, MO, USA, D6421) supplemented with 1% B27 (Life Technologies, Carlsbab, CA, USA), 0.5% N2 (Life Technologies), 10 ng mL^−1^ EGF, and 10 ng mL^−1^ b-FGF (PeproTech, Rocky Hill, CT, USA) (complete medium) under adherent conditions with laminin (R&D Systems, Minneapolis, MN, USA #3446-005-01). Cells were grown to confluence, dissociated using Accutase (Thermo Fisher Scientific, Waltham, MA, USA), and then split to convenience. We routinely used cultures expanded for no more than 15 passages.

### 2.2. Treatments

Synthetic peptides (>85% pure) were obtained from GenScript (Piscataway, NJ, USA). YGRKKRRQRRR was used as the TAT sequence, which enabled the cell penetration of peptides [44]. The TAT-Cx43_266-283_ sequence was TAT-AYFNGCSSPTAPLSPMSP (patent ID: WO2014191608A1). The TAT-Cx43_274-291_ sequence was TAT-PTAPLSPMSPPGYKLVTG. The peptides (TAT and TAT-Cx43_274-291_ as negative controls and TAT-Cx43_266-283_) were used at 50 μM in a culture medium at 37 °C for the indicated time.

Dasatinib (Selleckchem, Houston, TX, USA) was dissolved in dimethyl sulfoxide (DMSO) and used at 500 nM. Dasatinib or the corresponding volume of DMSO (vehicle) were added to the culture medium at 37 °C and incubated for the indicated time.

Chloroquine (CQ; Sigma, C6628) was dissolved in sterile distilled water and added to the culture medium at 37 °C at the indicated concentrations for the indicated times.

The starvation medium consisted of EBSS medium (11.6 mM NaCl, 5.4 mM KCl, 1.01 mM NaH_2_PO_4_·2H_2_O, 1.5 mM MgSO_4_·7H_2_O, 26 mM NaHCO_3_, 10 mg/mL Phenol Red, and 15 mM HEPES) supplemented with 14 mM glucose, and with EGF and b-FGF in the same concentration as the complete medium.

### 2.3. AlamarBlue Assay

AlamarBlue reagent (Bio-Rad, Hercules, CA, USA #BUF012) was used to determine cell viability. AlamarBlue was added to cells cultured at 37 °C at a final concentration of 5% *v*/*v* and incubated for 2 h. Fluorescence was measured (excitation 530–560 nm, emission 590 nm) in a microplate reader (Appliskan; Thermo Fisher Scientific).

### 2.4. Human Immunohistochemistry Microarrays

IHC samples and staining annotations (https://www.proteinatlas.org/about/assays+annotation#ih, accessed on 11 May 2021) were obtained from the Human Protein Atlas [45,46,47,48] (http://v20.proteinatlas.org, accessed on 11 May 2021). Briefly, staining levels were manually annotated by a specialist, followed by verification by a second specialist, and included evaluation of intensity levels, a fraction of stained cells, and (sub)cellular localization. For each protein analyzed, the annotations of all the samples were manually collected from the HPA portal, filtered for Age ≥ 18, and then the reported staining levels were represented as pie charts using R [49]. Glial cells (healthy donors) include those from the cortex, basal ganglia, and hippocampus (according to the HPA, non-neoplastic and morphologically normal cells). The reliability score (https://www.proteinatlas.org/about/assays+annotation#ih_reliability, accessed on 11 May 2021) for all the proteins selected for analysis was ‘enhanced’ or ‘supported,’ except for AMBRA1, whose reliability score was ‘approved.’ The collected annotations and patient data can be found in Tables S1–S9.

### 2.5. Immunofluorescence

Immunofluorescence was performed as previously described [10]. Briefly, cells were fixed in methanol for 10 min at −20 °C. The cells were then rinsed in phosphate-buffered saline (PBS) and incubated for 1 h in blocking solution (PBS containing 10% fetal calf serum (FCS), 0.1 M lysine, and 0.02% azide). The samples were incubated overnight at 4 °C with the indicated primary antibody prepared in blocking/permeabilization solution (with 0.1% Triton X-100): rabbit polyclonal antibody against LC3B (1:100; Cell Signaling Technology, Danvers, MA, USA #2775, RRID: AB_915950; although this antibody was directed against LC3B, cross-reactivity may exist with other LC3 isoforms according to the manufacturer), or mouse monoclonal antibody against p62 (1:100, Novus Biologicals, Centennial, CO, USA, H00008878-M01, clone 2C11, RRID: AB_548364). After repeated washes, they were incubated for 75 min with the corresponding secondary antibody prepared in blocking/permeabilization solution: anti-rabbit IgG or anti-mouse IgG Alexa Fluor 488-, Alexa Fluor 594-, or Alexa Fluor 647-conjugated antibodies (1:1000; Life Technologies). Finally, nuclear DNA was stained with 1 μg mL^−1^ 4′,6-diamidino-2-phenylindole (DAPI) for 1 min. Cells were mounted using ProLong mounting medium (Life Technologies) and imaged with a Stellaris 8 confocal microscope (Leica Microsystems, Wetzlar, Germany) with a pinhole aperture of 1 Airy Unit.

For quantification of LC3 and p62 punctae, images were analyzed using Fiji software. Six cells were randomly selected from each condition in each of 3 independent experiments. Then, the images were automatically thresholded to select LC3 or p62 punctae staining, and the integrated density (mean intensity × area) was plotted.

### 2.6. MTT Assay

MTT reagent (Fisher Scientific, Hampton, NH, 15214654) was used to determine cell viability [21]. Cells cultured at 37 °C were incubated in the dark for 75 min with a culture medium containing 0.5 mg mL^−1^ MTT. The cells were then carefully washed with PBS once and incubated for 10 min in the dark in DMSO with mild shaking. Absorbance was measured at a wavelength of 570 nm using a microplate reader (Appliskan; Thermo Scientific).

### 2.7. Patient Survival Analysis

GBM data for protein expression of c-Src pY416 and pY527 were downloaded from the TRGAted portal [50] (https://nborcherding.shinyapps.io/TRGAted, accessed on 27 May 2021). These data were obtained by The Cancer Proteome Atlas [51] with reverse-phase protein arrays (RPPAs), a high-throughput approach to protein quantification based on antibodies, performed for 200 proteins on samples from The Cancer Genome Atlas [52] (https://www.cancer.gov/tcga, accessed on 11 May 2021). The downloaded TRGAted data were plotted in R [49] (version 4.0.3) using the ‘survival’ and ‘survminer’ packages, as performed by TRGAted [50]. The optimal cut-off for grouping according to protein expression was determined with the ‘surv_cutpoint’ function of the ‘survminer’ package, which used the maximally selected rank statistic, as performed by TRGAted [50]. The hazard ratio refers to the probability of dying by the next time point of a patient in the low expression group compared to a patient in the high expression group. The log-rank *p*-value refers to the probability that the risk of death (hazard) is the same in both groups. The Kaplan–Meier curves shown correspond to progression-free survival.

### 2.8. Time-Lapse Microscopy (Live-Cell)

As previously described [21], GSCs were seeded in 12-well plates for time-lapse microscopy. Cells were cultured in starvation medium (see Treatments) for 24 h and then, the indicated treatments at the indicated concentrations were added to the medium and the cells were allowed to equilibrate for 1 h in the microscope incubator before imaging. The cells were recorded for 48 h. Then, the medium was replaced with a complete medium without treatment, and the cells were recorded for a further 48 h. In all videos, phase-contrast microphotographs of each experimental condition were taken every 10 min for live-cell imaging with an inverted Zeiss Axio Observer Z1 microscope coupled to an AxioCam MRm camera. The system included an automated XY stage controller and a humidified incubator set at 37 °C and 5% CO_2_.

### 2.9. Transmission Electron Microscopy

Transmission electron microscopy was performed as previously reported [21]. Cell culture preparations were fixed in 2% formaldehyde and 2% glutaraldehyde in phosphate buffer for 30 min at 4 °C. Samples were then post-fixed with 1% osmium tetroxide in water, dehydrated through a graded ethanol series, and embedded in Epoxy EMbed-812 resin (Electron Microscopy Sciences, Hatfield, PA, USA). Ultrathin sections were obtained with a Leica EM UC7 ultramicrotome, contrasted with uranyl acetate and lead citrate, and analyzed using a Tecnai Spirit Twin 120 kV electron microscope with a CCD Gatan Orius SC200D camera with DigitalMicrograph™ software. Procedures were performed at the Electron Microscopy Facilities-NUCLEUS of the University of Salamanca.

### 2.10. Western Blotting

Western blotting was performed as described previously [14]. Briefly, equal amounts of proteins across conditions were separated on NuPAGE Novex Bis-Tris 4–12% Midi gels (Life Technologies) at room temperature and constant voltage. Proteins were transferred to a nitrocellulose membrane (iBlot Gel Transfer Stacks Nitrocellulose) using an iBlot dry blotting system (Life Technologies). After blocking, the membranes were incubated overnight at 4 °C with primary antibodies: mouse monoclonal antibody against glyceraldehyde phosphate dehydrogenase (GAPDH; 1:5000; Thermo Fisher Scientific AM4300, RRID: AB_437392), or rabbit polyclonal antibody against LC3B (1:100; Cell Signaling Technology #2775, RRID: AB_915950, although this antibody was directed against LC3B, cross-reactivity may exist with other LC3 isoforms according to the manufacturer). After extensive washing, the membranes were incubated with peroxidase-conjugated anti-mouse IgG or anti-rabbit IgG antibodies (1:5000; Jackson ImmunoResearch, Cambridgeshire, United Kingdom) and developed with a chemiluminescent substrate (Western Blotting Luminol Reagent; Santa Cruz Biotechnology, Dallas, TX, USA) in a MicroChemi imaging system (Bioimaging Systems, Upland, CA, USA). Uncropped blots are shown in Figure S1. GAPDH was used as a loading control.

### 2.11. Statistical Analysis

Results were expressed as the means ± s.e.m. The number of technical replicates and independent experiments was indicated for each experiment in its corresponding figure or figure legend and was determined according to the previous experience of the research group. For comparison between 2 groups, data were analyzed by a two-tailed Student’s *t*-test. When more than 2 groups were compared, data were analyzed by one-way ANOVA, and confidence intervals (95%) and significance were corrected for multiple comparisons with the Tukey test. In all cases, values were considered significant when *p* < 0.05.

## 3. Results

### 3.1. c-Src Activity Is a Relevant Target in Glioblastoma Patients

**Figure 1:**
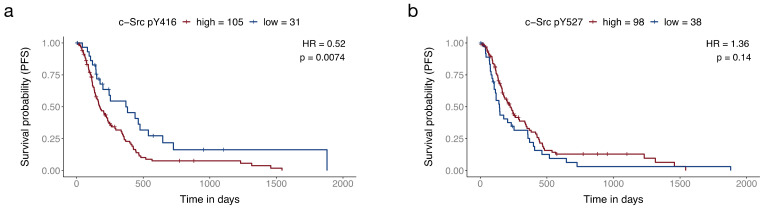
Decreased c-Src activity correlates with better survival in GBM patients. The optimal expression cut-off was determined by TRGATed using maximally selected rank statistics. The hazard ratio (HR) refers to the probability of dying by the next time point of a patient in the low expression group compared to a patient in the high expression group. The log-rank *p*-value refers to the probability that the risk of death (hazard) is the same in both groups. Progression-free survival (PFS) (Kaplan–Meier curves) of GBM patients divided according to c-Src pY416 ((**a**); active c-Src form) or pY527 ((**b**); inactive c-Src form) levels.

In our previous studies, we have shown that TAT-Cx43_266-283_ inhibits the oncogenic activity of c-Src through the recruitment of its physiological inhibitors, PTEN and CSK [9,10,14]. The relevance of c-Src in GBM has been explored by us and others through various approaches, using in vitro and in vivo systems, as well as patient-derived samples. Indeed, molecular, cellular, and preclinical data strongly suggest that c-Src activity inhibition could benefit GBM patients [14,15,21,53,54,55]. However, to our knowledge, the relationship between key c-Src phosphorylation levels (i.e., activity) and GBM patient survival remains unexplored. Hence, we took advantage of protein expression data obtained with reverse-phase protein arrays from glioblastoma patients performed by The Cancer Proteome Atlas (https://tcpaportal.org/tcpa/, accessed on 11 May 2021) [51] available via the TRGTAted portal (https://nborcherding.shinyapps.io/TRGAted/, data downloaded on 10 November 2017) [50]. The analysis revealed that lower activity of c-Src (indicated by lower phosphorylation levels of pY416) correlated with better progression-free survival (Figure 1a). We also analyzed phosphorylation levels of pY527 Src, an inactive form of c-Src, and we found that higher levels of pY527 Src correlate with better progression-free survival, although this correlation was not statistically significant (Figure 1b). Patients with low activity of c-Src (lower pY416 c-Src levels) were almost twice as likely to survive in any given time point than those with high activity of c-Src (pY416 c-Src levels), as indicated by the hazard ratio (0.52). These results, together with previous studies, argue in favour of c-Src inhibition as a therapeutic strategy in GBM.

### 3.2. TAT-Cx43_266-283_ Blocks Basal Autophagic Flux in GSCs

**Figure 2:**
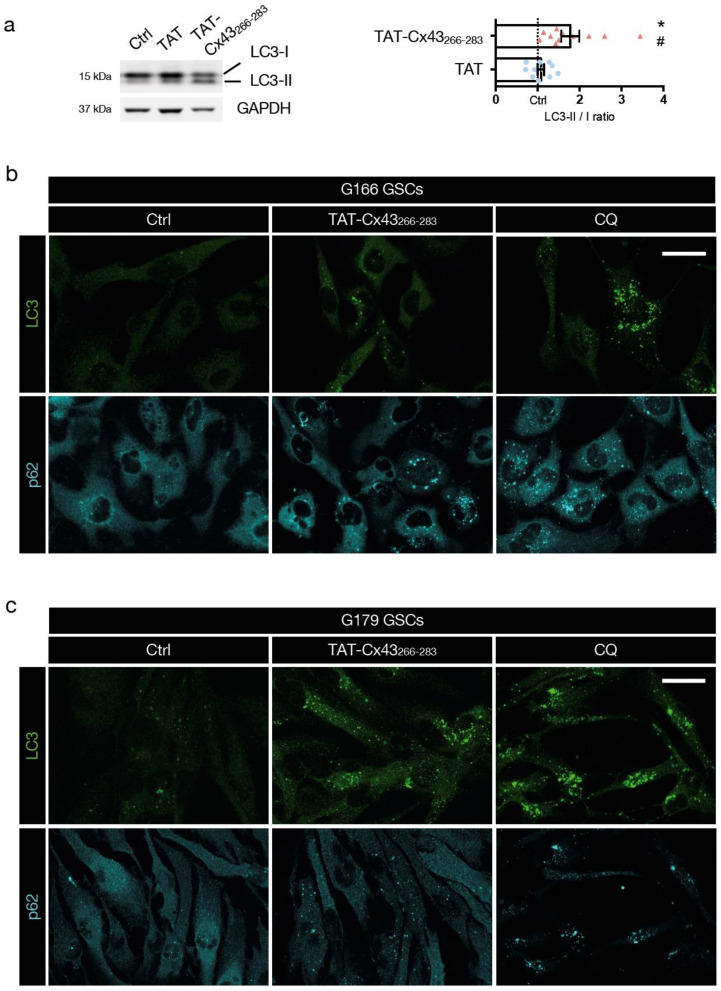
TAT-Cx43_266-283_ blocks basal autophagic flux in GSCs. GSCs were treated with the indicated molecules in complete medium. (**a**) LC3-I and II levels in G166 GSCs detected by Western blotting. Figure S1 shows the complete blots. The plot shows the ratio of LC3-II to LC3-I normalized to the untreated control of the same experiment (dotted line). Data are mean ± s.e.m. (* *p* < 0.05 vs. control; # *p* < 0.05 vs. TAT). Each point represents an independent biological replicate. (**b**,**c**) Representative confocal microscopy images of the effect of TAT-Cx43_266-283_ (24 h, 50 μM) and CQ (4 h, 100 μM) on G166 GSCs (**b**) or G179 GSCs (**c**) autophagic flux assessed by LC3 and p62 immunofluorescence. Note the increase in LC3 and p62 vesicles in TAT-Cx43_266-283_-treated GSCs, indicative of autophagosome accumulation and impaired autophagic flux. Scale bars: 10 (**b**) and 50 (**c**) μm. Similar results were obtained in at least three independent biological replicates.

Once established the potential benefit of c-Src inhibition in GBM and considering the pro-tumoral role of autophagy in established tumors, we decided to investigate the role of TAT-Cx43_266-283_, a c-Src inhibitor, in GSC autophagy. We first evaluated conversion of LC3-I (cytoplasmic) to LC3-II (autophagosome-bound) in basal conditions (complete medium) by Western blotting, as LC3-II remains on mature autophagosomes until fusion with lysosomes is completed [26,56]. We found that TAT-Cx43_266-283_ increased the ratio of LC3-II/I compared to the untreated and TAT controls (Figure 2a and Figure S1). The increased LC3-II/I ratio can result from either up-regulation of autophagosome formation or blockage of autophagic degradation [57]. To discriminate between these two scenarios, we investigated GSC autophagic flux by immunofluorescence of LC3 and p62 (which accumulates in cells when autophagy is inhibited [58]). CQ was included as a positive control of autophagy blockage [39]. Although there was single-cell-level heterogeneity, both CQ (4 h, 100 μM) and TAT-Cx43_266-283_ (24 h, 50 μM) induced accumulation of LC3 and p62 puncta in the cytoplasm of G166 and G179 GSCs (Figure 2b,c, respectively), revealing blocked autophagic flux in GSCs.

**Figure 3:**
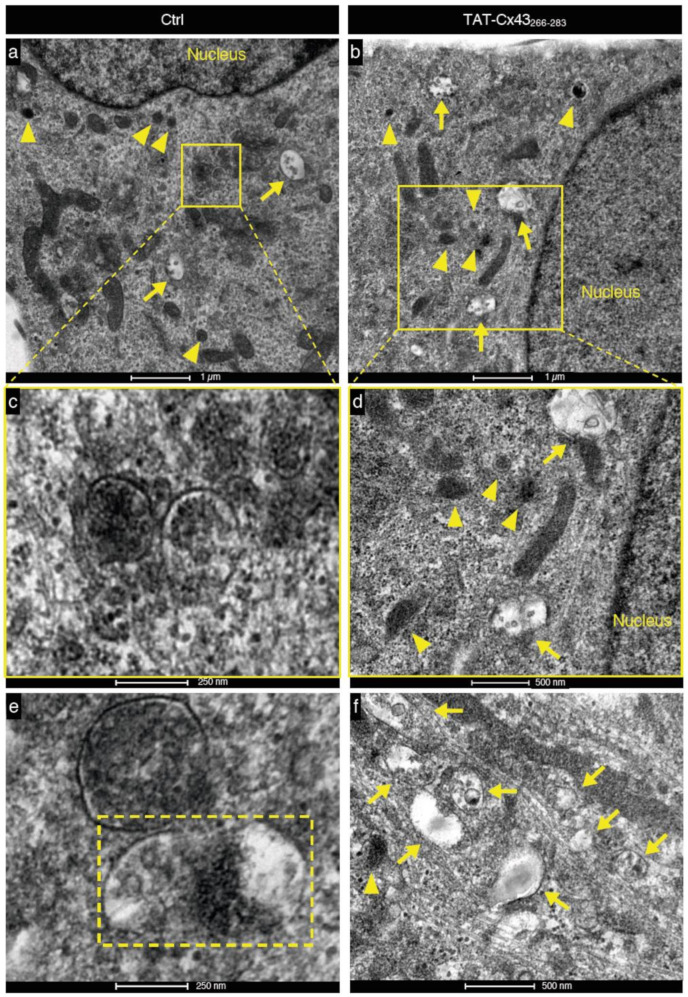
Blockage of the basal autophagic flux by TAT-Cx43_266-283_ in GSCs is also detected by transmission electron microscopy. (**a**,**b**) Representative transmission electron microscopy images showing lysosomes (arrowheads) and autophagosomes (arrows), including cellular contents in both control (**a**) and TAT-Cx43_266-283_-treated GSCs (**b**). (**c**,**e**) Autophagolysosomes, i.e., autophagosomes fused to lysosomes, were detected in control GSCs (**a**,**c**,**e**) but not in TAT-Cx43_266-283_-treated GSCs (**b**,**d**,**f**). (**c**) Magnification of the autophagolysosomes shows the denser material, indicating that digestion of the contents is occurring. (**d**) Autophagosomes and lysosomes were found in TAT-Cx43_266-283_-treated GSCs, but no fusion events were detected. (**e**) Lysosomes and autophagosomes undergoing fusion were observed in the control GSCs but not in TAT-Cx43_266-283_-treated GSCs (rectangle with discontinuous line). (**f**) Accumulation of autophagosomes in TAT-Cx43_266-283_-treated GSCs.

To confirm these results, we evaluated autophagy using transmission electron microscopy (TEM), another technique widely used for studying autophagic processes [58]. In TEM images, autophagosomes have a double membrane and a clear or distinct content, whereas lysosomes present a single membrane and very electron-dense homogeneous or heterogeneous content, indicative of ongoing degradation. We found an abundance of characteristic autophagosomes (arrows) and lysosomes (arrowheads) in both untreated and TAT-Cx43_266-283_ treated GSCs (Figure 3), suggesting that biogenesis of these compartments is frequent in both conditions and not compromised by TAT-Cx43_266-283_. We found many instances of autophagosome-lysosome fusions in untreated G166 GSCs (Figure 3a,c,e), but not in TAT-Cx43_266-283_-treated GSCs. In contrast, accumulations of autophagosomes, sometimes in proximity to lysosomes, were found in TAT-Cx43_266-283_ GSCs (Figure 3b,d,f) but not in control GSCs. Taken together, these findings suggest that TAT-Cx43_266-283_ induces the blockage of late events in basal autophagy in GSCs, leading to an accumulation of autophagosomes.

### 3.3. TAT-Cx43_266-283_ Increases Cell Death in Nutrient-Deprived Dormant GSCs

**Figure 4:**
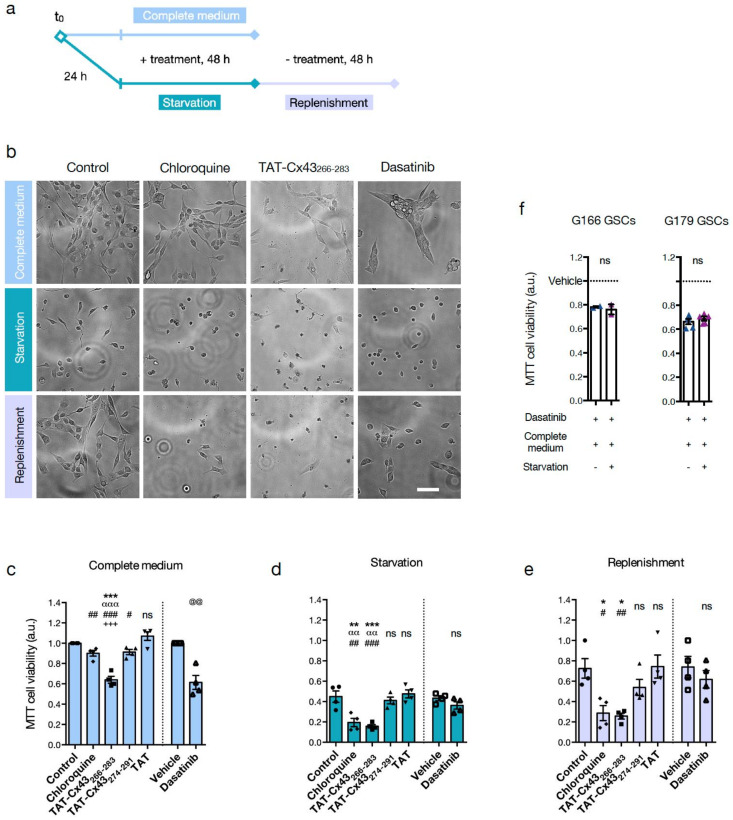
TAT-Cx43_266-283_ causes cell death in nutrient-deprived dormant GSCs. (**a**) Scheme of the experimental design for (**b**-**e**). Treatment concentrations were: 50 μM cell-penetrating peptides, 500 nM dasatinib, 50 μM CQ. (**b**-**e**) are G166 GSCs. (**b**) Representative cell culture fields were imaged with an EVOS Floid imaging station at the endpoint of the indicated conditions and treatments. Scale bar: 100 μm. (**c**-**e**) MTT assays were used to assess cell viability in the indicated conditions and treatments. Data were normalized to the control of the complete medium. (**f**) MTT assay of G166 GSCs and G179 GSCs. GSCs were treated with dasatinib (1 μM) for 24 h in complete medium, and then the medium was replaced with starvation medium or complete medium also containing dasatinib (1 μM) for another 24 h. Data were normalized to the vehicle of each condition. All data are mean ± s.e.m. from at least three independent experiments. Each data point is the average of at least two technical replicates. * *p* < 0.05, ** *p* < 0.01, *** *p* < 0.001 vs. control; αα *p* < 0.01, ααα *p* < 0.001 vs. TAT-Cx43_266-283_; # *p* < 0.05, ## *p* < 0.01, ### *p* < 0.001 vs. TAT; +*++ p* < 0.001 vs. CQ; @@ *p* < 0.01 vs. vehicle; ns, not significant. See also related Figure S2 and Videos S1–S4.

To delve into the functional role of autophagy in GSC dormancy, we examined the impact of TAT-Cx43_266-283_ on cell viability in basal conditions (cells cultured in complete medium) and after the induction of dormancy by nutrient starvation (Figure 4a). The impact of four other treatments was also examined to help elucidate the underlying mechanisms: CQ, to identify autophagy as a survival mechanism; two peptides as negative controls, TAT and TAT-Cx43_274-291_ [9,10,14]; and positive control of c-Src involvement, dasatinib, an inhibitor of c-Src activity [59]. As illustrated in Figure 4a, GSCs were treated in a complete medium for 48 h. In parallel, GSCs were starved for 24 h to induce dormancy and then treated for 48 h. Then, GSCs were switched back to complete medium (replenishment) for a further 48 h, without treatment, to revert dormancy and resume cell proliferation. Live cell time-lapse videos from G179 and G166 GSCs were recorded to follow changes in cell phenotype (Supplementary Videos S1–S4), and representative images are shown in Figure 4a. Moreover, cell viability was assessed with MTT (Figure 4c–e) and with Alamar Blue (AlaB) (Figure S2).

Our results indicate that, in complete medium, CQ did not have a large effect on GSC viability, as opposed to TAT-Cx43_266-283_ and dasatinib (as previously reported [10,21,22]) (Figure 4b,c, and Figure S2), suggesting that GSCs do not rely strongly on autophagy when nutrients are widely available from the environment. However, after induction of dormancy by nutrient starvation, both TAT-Cx43_266-283_ and CQ decreased GSC viability and caused massive cell death compared to control, TAT or TAT-Cx43_274-291_ (Figure 4b,d and Figure S2 and Videos S1–S4), suggesting that TAT-Cx43_266-283_ inhibits autophagy as CQ does, and that autophagy is necessary for dormant GSC survival. It should be noted that untreated G166 GSCs, under nutrient deprivation, adopt a round and slightly motile, dormant-like morphology (Video S1a and Figure 4b), which is very different from the highly vacuolated cytoplasm and non-motile appearance of TAT-Cx43_266-283_-treated G166 GSCs under nutrient deprivation (Video S2a and Figure 4b). Similar videos were obtained with untreated and TAT-Cx43_266-283_-treated G179 GSCs (Videos S3a and S4a, respectively). Interestingly, dasatinib did not decrease GSC viability or induce cell death in this condition. To examine this finding further, GSCs were first treated with dasatinib in a complete medium, and then the medium was replaced with a starvation medium (or complete medium for reference) containing dasatinib. We found that dasatinib did not enhance G166 or G179 GSC death in starvation compared to complete medium (Figure 4f) in contrast with the effect found with TAT-Cx43_266-283_ [21]. These results suggest that c-Src inhibition, at least with dasatinib, is not sufficient to disrupt GSC dormancy, but we cannot exclude c-Src participation. Finally, upon nutrient replenishment for 48h, TAT, TAT-Cx43_274-291_, and dasatinib-treated GSCs resumed cell attachment, motility, and proliferation, similarly to their respective controls (Figure 4b,e and Figure S2, Video S1b for untreated G166 GSCs and 3b for untreated G179 GSCs), yet CQ and TAT-Cx43_266-283_-treated GSCs maintained a lower cell attachment, motility, proliferation and cell viability (Figure 4b,e and Figure S2, Video S2b for TAT-Cx43_266-283_-treated G166 GSCs and 4b for TAT-Cx43_266-283_-treated GSCs G179 GSCs), confirming the increased cell death of dormant GSCs after CQ and TAT-Cx43_266-283_ treatment.

### 3.4. TAT-Cx43_266-283_ Blocks Autophagy in Nutrient-Deprived Dormant GSCs

**Figure 5:**
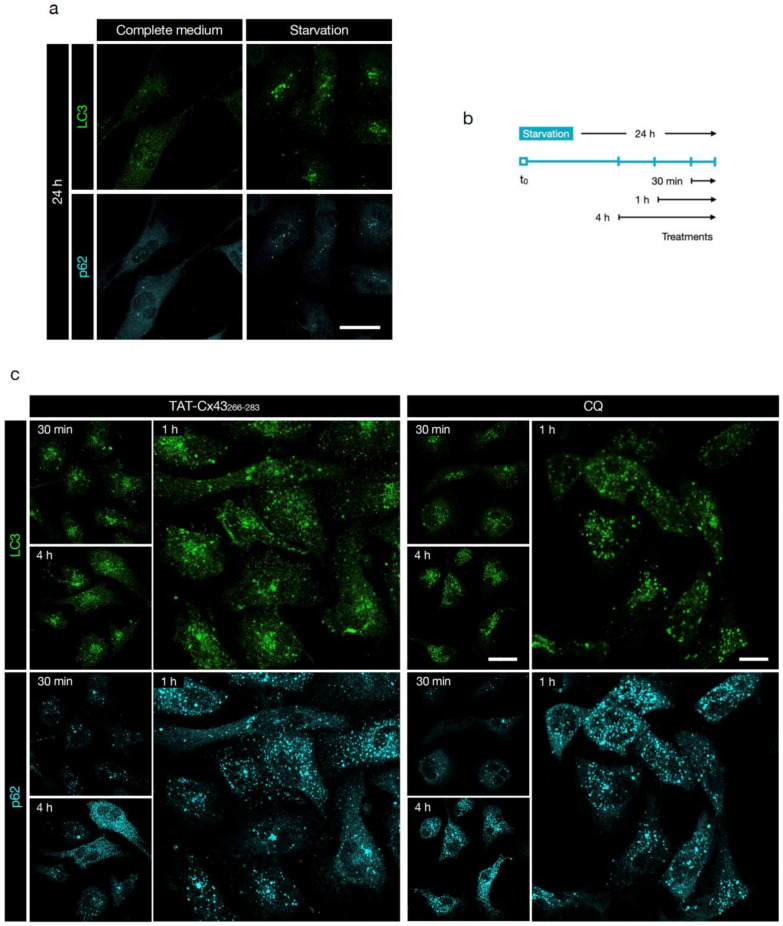
TAT-Cx43_266-283_ blocks autophagy in nutrient-deprived dormant GSCs. Images were acquired by confocal microscopy. (**a**) Starvation induces autophagy in G166 GSCs, as evidenced by the increased number and intensity staining of LC3 vesicles and decreased number of vesicles and intensity staining of p62. Scale bar: 50 μm. (**b**) Scheme of the experimental design. G166 GSCs were fixed at the indicated time points, and autophagic flux was assessed by immunofluorescence analysis of LC3 and p62 staining. (**c**) Representative images of the effect of TAT-Cx43_266-283_ (50 μM) and CQ (50 μM) on the autophagic flux of starvation-induced dormant G166 GSCs, assessed by LC3 and p62 immunofluorescence. Note the increase in LC3 and p62 vesicles in TAT-Cx43_266-283_-treated and CQ-treated GSCs, indicative of autophagosome accumulation and impaired autophagic flux. Scale bar: 50 μm (30 min, 4 h), 25 μm (1 h). Similar results were obtained in at least three independent biological replicates. See also related Figure S3 for quantification of these data.

To ascertain whether inhibition of autophagy participated in the effect of TAT-Cx43_266-283_ in dormant GSCs, we performed immunofluorescence analysis of LC3 and p62 in nutrient-starvation-induced dormant GSCs. First, we corroborated that autophagy was activated after nutrient starvation, as evidenced by the increased number and intensity staining of LC3 vesicles and the decreased number of vesicles and intensity staining of p62 (Figure 5a, quantification in Figure S3a), indicative of high autophagic flux. Next, we examined autophagic flux in nutrient starvation conditions after the addition of TAT-Cx43_266-283_ or CQ for different time durations (Figure 5b) prior to cell analysis. We found that both treatments induced accumulation of LC3 and p62 vesicles (Figure 5c, quantification in Figure S3b), confirming the blockage at the late stage of autophagy. Whereas in complete medium TAT-Cx43_266-283_ effect on the autophagic flux was milder than that of CQ (Figure 2b), in starvation the effect of these two molecules was very similar (Figure 5c). These results indicate that TAT-Cx43_266-283_ causes autophagosome accumulation suggestive of arrested autophagic flux after nutrient starvation-induced dormancy in GSCs.

### 3.5. Autophagy Mediators Are Over-Expressed at the Protein Level in High-Grade Glioma Patients

**Figure 6:**
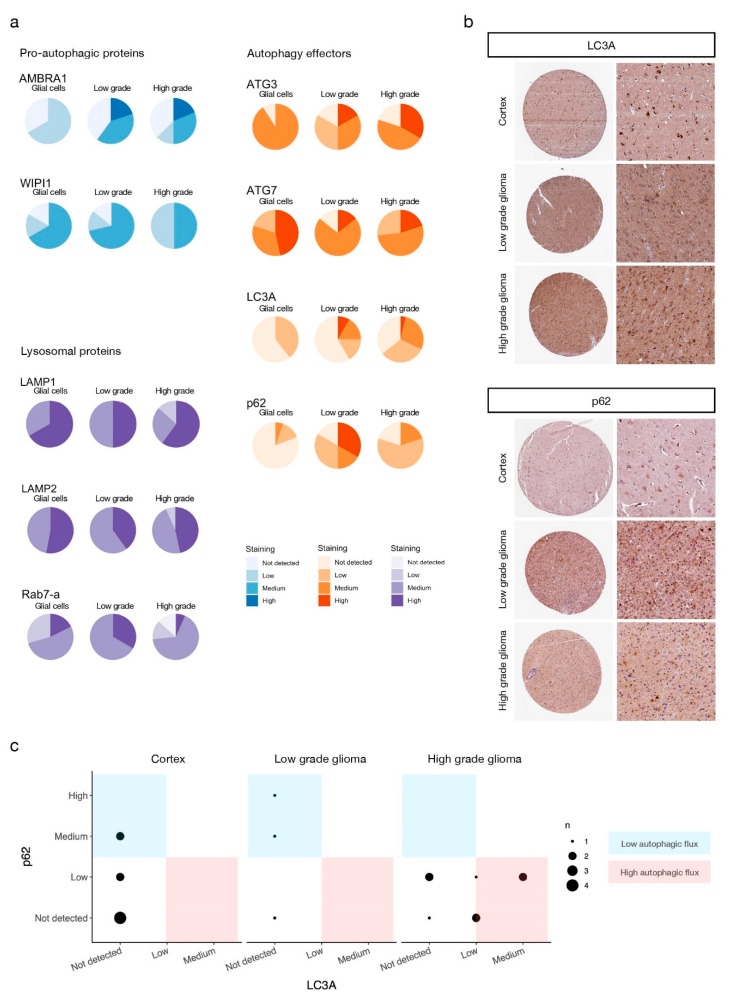
Autophagy mediators are over-expressed at the protein level in high grade glioma patients. (**a**) Staining levels of the indicated proteins evaluated by IHC of human healthy brain, low-grade, and high-grade glioma tissue microarrays available from the HPA (see Materials and Methods). Antibodies used for staining and patient data can be found in Tables S1–S9. IHC staining levels were manually annotated by the HPA specialists. (**b**) Representative IHC microarrays from the HPA (https://www.proteinatlas.org, accessed on 11 May 2021) stained for the indicated proteins. Staining levels of selected samples for LC3A: cortex–low staining (glial cells), low- and high-grade glioma–medium staining. Staining levels of selected samples for p62: cortex–not detected (glial cells), low-grade glioma–high staining, high-grade glioma–low staining. (**c**) Autophagic flux of IHC samples (see main text and Materials and Methods for details).

Because our results revealed that autophagy is necessary for GSC survival under nutrient deprivation and that TAT-Cx43_266-283_ arrests autophagic flux, resulting in cell death, we assessed the role of autophagy in a more clinical setting. To do so, we took advantage of the publicly available protein expression data from the Human Protein Atlas [45,46,47,48] (HPA, https://www.proteinatlas.org, accessed on 11 May 2021). The HPA provides access to immunohistochemistry of thousands of proteins performed on tissue microarrays from healthy and cancer donors that are manually annotated for staining and reliability, taking into consideration inter-antibody staining pattern consistency, RNA-protein expression consistency, and (sub)cellular localization of staining, and classified by staining levels by professional specialists according to fixed guidelines (see Materials and Methods). We analyzed the staining of 3 groups of well-known autophagy-related proteins in healthy tissue (glial cells from brain cortex, hippocampus, and caudate) and low and high grade (which includes GBM) glioma tissue: pro-autophagic proteins AMBRA1 [60] and WIPI1 [61], autophagy effectors ATG3, ATG7, LC3A and p62, and lysosomal proteins LAMP1, LAMP2, and Rab7-a. While the expression of lysosomal proteins did not change greatly across tissue types, both pro-autophagic proteins and autophagy effectors (with the exception of ATG7) showed increased staining in tumoral tissues (Figure 6a,b).

The autophagy effector p62, in contrast with most proteins evaluated here, showed stronger staining in low-grade glioma compared to healthy tissue and high-grade glioma. However, LC3A staining in low-grade glioma was similar to that of high-grade glioma (Figure 6a,b). This prompted us to classify the samples according to their autophagic flux to evaluate differences between low and high-grade glioma. Autophagic flux was estimated as a ratio between LC3A and p62 expression: high autophagic flux corresponds to higher LC3A and lowers p62 expression, whereas low autophagic flux corresponds to lower LC3A and higher p62 expression [32,58]. This analysis was only possible in a limited number of samples for which the expression of both LC3A and p62 was evaluated in the same donor with the same antibodies. Despite the small sample size, the results showed that healthy tissue and low-grade gliomas tend to exhibit lower autophagic flux than high-grade gliomas (Figure 6c), in accordance with previous studies [31,32].

## 4. Discussion

Autophagy is a complex physiological process that, among other important roles, can be responsible for cancer cell survival during dormancy. Our previous study showed that the antitumor activity of the c-Src inhibitor peptide, TAT-Cx43_266-283_, includes the impairment of GSC dormancy in nutrient-deprived conditions [21]. Because of the prominent role played by dormancy in cancer and the role of autophagy in sustaining dormancy, in this study, we explored the contribution of autophagy to the antitumor effects of TAT-Cx43_266-283_ in GSCs.

We found that TAT-Cx43_266-283_ induces the accumulation of autophagosomes in GSCs, limiting the late stages of the autophagic flux. Indeed, this effect is similar to that found with CQ, a well-known inhibitor of late events in autophagy [39]. The sole accumulation of autophagosomes can induce cytotoxicity in some cells [62]. However, although TAT-Cx43_266-283_ reduced GSC viability in complete medium, the effect of CQ was very mild and only detectable with one of two viability assays, suggesting that most GSCs can survive the effects of autophagy blockage when nutrients are plentiful, in agreement with previous results in glioma [63]. Because dasatinib reduced GSC viability in complete medium in a similar way to TAT-Cx43_266-283_, we propose that the effect of TAT-Cx43_266-283_ on GSC survival in complete medium is due to c-Src inhibition, as previously shown [14,21]. When GSCs are nutrient-deprived, autophagy becomes necessary for cell survival, as evidenced by the detrimental effect of CQ on cell viability, an outcome compatible with cytotoxicity due to accumulation of autophagosomes and the subsequent lack of nutrients derived from autophagy. The effect of TAT-Cx43_266-283_ on GSC death was stronger under nutrient deprivation than that found in complete medium, suggesting that blocking the autophagic flux contributes to the antitumor effects of this compound under nutrient starvation.

We hypothesized that TAT-Cx43_266-283_ might block the autophagic flux through the inhibition of c-Src. Indeed, it was recently shown that lysosomal c-Src decreases LC3-II levels and autophagic flux in a kinase activity-dependent manner and that lysosomal c-Src and other Src family kinases promote autophagosome-lysosome fusion to maintain viability under nutrient starvation [64]. Surprisingly, dasatinib, a c-Src inhibitor, did not affect GSC survival under nutrient deprivation, indicating that the effect of dasatinib on other signaling pathways, including other members of the Src kinase family, BCR-ABL, c-KIT, PDGFR, and ephrin A2 [65], might be counteracting the effect on autophagy or that c-Src inhibition might not be sufficient to promote cell death in dormant GSCs. In agreement with the last option, in dormant breast cancer cells, c-Src inhibition is necessary but not sufficient to induce cell death; instead, combined c-Src and ERK1/2 inhibition is required to induce apoptosis [66]. In fact, ERK activity is regarded as a dormancy regulator [67,68,69], and there is bidirectional crosstalk between ERK and autophagy [70,71,72,73]. Another mechanism underlying autophagosome accumulation due to TAT-Cx43_266-283_ could be c-Src or PTEN-dependent modulation [9,14] of the mTOR/Pi3K/AKT pathway. Indeed, our previous studies showed that TAT-Cx43_266-283_, in addition to inhibiting c-Src activity, upregulates PTEN levels and reduces Akt activity [9,14]. Several works have explored the regulation of autophagy and cell death orchestrated by this axis [63,74,75,76,77], reaching the overarching conclusion that autophagy can be manipulated through these pathways to induce cell death, although sometimes inhibition of additional pathways, notably autophagic flux itself, is needed, once again speaking to the context-dependent effects of autophagy. Therefore, we suggest that the inhibition of c-Src promoted by TAT-Cx43_266-283_ might be required for the inhibition of the autophagic flux, but other signaling pathways might also be involved. In this regard, the sequence 266-283 in Cx43 interacts with mitogen-activated protein kinases (MAPKs) [78], which are known to regulate autophagy [70,72]. Therefore, the participation of additional c-Src-independent pathways in the effect of TAT-Cx43_266-283_ in dormant GSCs is plausible and deserves further exploration to reach a deeper understanding of the mechanism of action of TAT-Cx43_266-283_.

One of the reasons for which clinical outcomes of successful preclinical antitumor drugs fail is because a subset of tumoral cells develops survival mechanisms, such as autophagy, to resist challenging conditions [79]. In this study, we reveal that TAT-Cx43_266-283_ impairs autophagy in GSCs, which can acquire a dormancy state and drive tumor relapse after chemotherapy [37,80]. Our results confirmed that autophagy, which can act as an internal source of nutrients, is a crucial process required for GSC survival in starvation conditions. Starvation promotes a dormancy-like state in GSCs, which is reverted upon nutrient replenishment [21]. Importantly, autophagy inhibition by TAT-Cx43_266-283_ or by the classical inhibitor, CQ, promoted GSC death under nutrient deprivation, preventing the proliferative response to nutrient replenishment that did occur in the absence of CQ or TAT-Cx43_266-283_. Importantly, we found that TAT-Cx43_266-283_ reduces GSC survival both in a nutrient-complete or -deprived environment, while CQ and dasatinib strongly depend on the nutrient availability to exert their effects.

As previously introduced, several studies have addressed the impact of autophagy on the clinical outcome of gliomas or GBM through mRNA or protein expression analysis and concluded that increased autophagic flux correlates with higher glioma grade and worse patient survival [31,32]. In agreement with this, our study of the levels of autophagy-related proteins in databases with samples from glioma patients indicates that autophagic activity is upregulated in glioblastoma as compared to low-grade gliomas or healthy tissue, supporting the relevance of autophagy for the progression of glioblastoma and the interest of targeting this pathway. However, given that autophagy is an intricate process with context-dependent consequences in health and disease, it is possible that arresting the autophagic flux could have detrimental effects in certain situations. For instance, p62 has a myriad of roles beyond binding autophagic cargo, such as functions in adipogenesis, the anti-oxidative response, apoptosis, inflammation, or nutrient sensing (reviewed in [81]). In fact, in some tumor models, p62 accumulation has been found to promote tumor growth through the NF-KB pathway [82,83]. Moreover, alternative energy-scavenging strategies, including chaperone-mediated autophagy, an autophagosome-independent form of autophagy, can procure amino acids for protein synthesis and gluconeogenesis (reviewed in [83]). It is important to study these potential side issues as they can underlay resistance to TAT-Cx43_266-283_ and, hence, represent targets for combination therapy to enhance the peptide’s antitumor effect.

Autophagy and cell metabolism are intimately connected and modulate each other [84,85,86]. To sustain energy demands, nutrient starvation can induce dependence on autophagy, and, conversely, autophagy blockage can enhance dependence on glycolytic or mitochondrial metabolism. Importantly, TAT-Cx43_266-283_ was capable of decreasing glucose metabolism, metabolic plasticity [21], and autophagic flux, resulting in a potent antitumor effect independently of the nutrient environment. In the same conditions, dasatinib and CQ, two drugs approved for human glioblastoma therapy with unsuccessful outcomes [42,43,87], failed to exhibit the same effect. Our study shows that these drugs, contrary to TAT-Cx43_266-283_, depend on specific (nutrient) environments to promote GSC death, as has been previously reported for other antitumor drugs [88]. These findings, together with the wide-spectrum antitumor properties and the cell-selectivity of TAT-Cx43_266-283_, which does not exhibit detrimental effects on healthy brain cells [9,10,14,21,22], further argues in favor of the potential efficacy of TAT-Cx43_266-283_ in the clinical context.

## 5. Conclusions

The inhibition of the autophagic flux contributes to the antitumor effects of TAT-Cx43_266-283_ in GSCs.

TAT-Cx43_266-283_ leads to cell death in nutrient starvation-induced dormant GSCs, a GSC subset that can drive relapse after therapy.

TAT-Cx43_266-283_ exerts its antitumor effect both in nutrient-complete and nutrient-deprived environments, which constitutes an advantage over other unsuccessful drugs against GBM.

Patient data analysis based on human samples from the Human Protein Atlas suggests that GBM exhibits enhanced autophagic flux compared to healthy and low-grade glioma tissues, arguing in favor of autophagy inhibition as a therapy strategy.

## 6. Patents

No new patents result from the work reported in this manuscript.

## Data Availability

GBM patient survival data is available at https://nborcherding.shinyapps.io/TRGAted (accessed on 27 May 2021). IHC of human microarrays is available at http://v20.proteinatlas.org (accessed on 11 May 2021).

## Supplementary Materials

The following are available online at www.mdpi.com/2072-6694/13/17/4262/s1, Figure S1: Uncropped Western blots, Figure S2: Viability of nutrient-deprived dormant G166 GSCs, Tables S1–S9: Annotations and patient data of IHC microarrays, Videos S1–S4: Nutrient starvation-induced dormant GSCs recorded by time-lapse microscopy.
