# Supplementary material for: Impairment of Autophagic Flux Participates in the Antitumor Effects of TAT-Cx43266-283 in Glioblastoma Stem Cells
Source: Cancers (Basel). 2021 Aug 24;13(17):4262. doi: 10.3390/cancers13174262 (PMC8428230; doi:10.3390/cancers13174262)
Supplement: Supplementary file 1 [file cancers-13-04262-s001.zip › supplementary information/Supplementary information.pdf]

Fig. S1

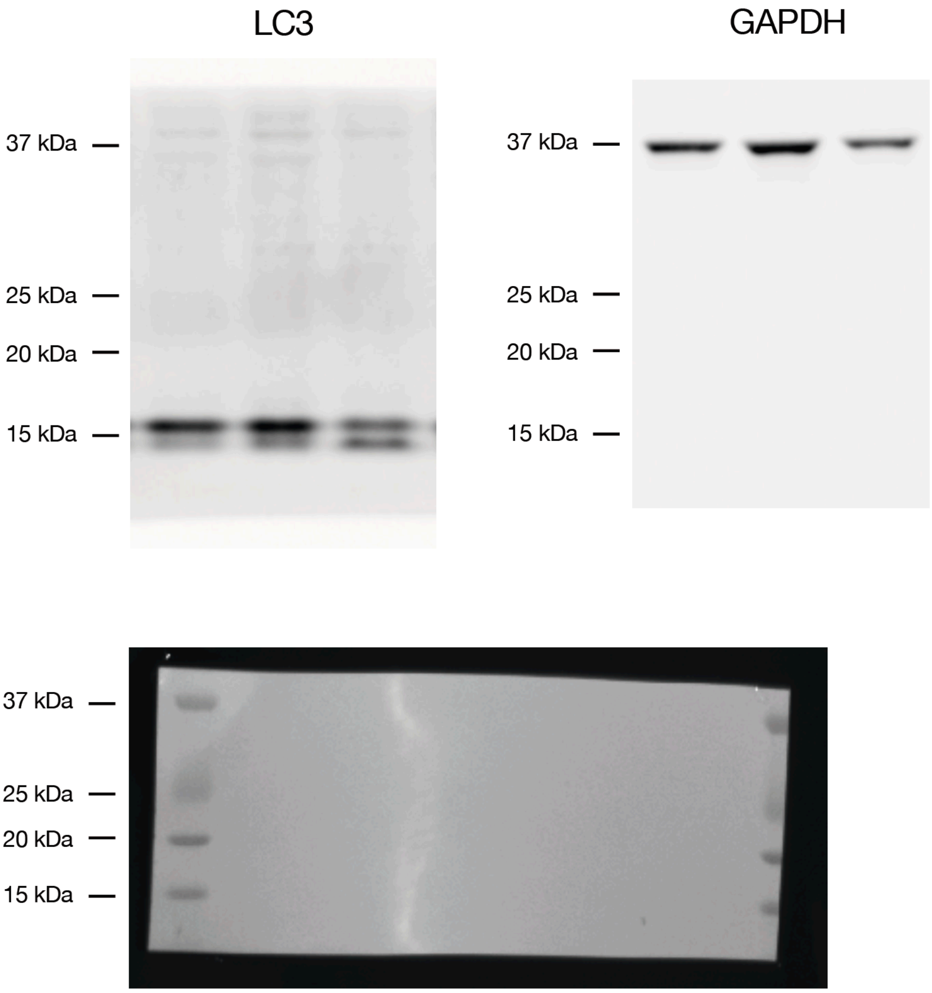

Figure S1. Uncropped western of LC3 and GAPDH (Fig. 2 a). The bottom image shows the blot with molecular weight markers.

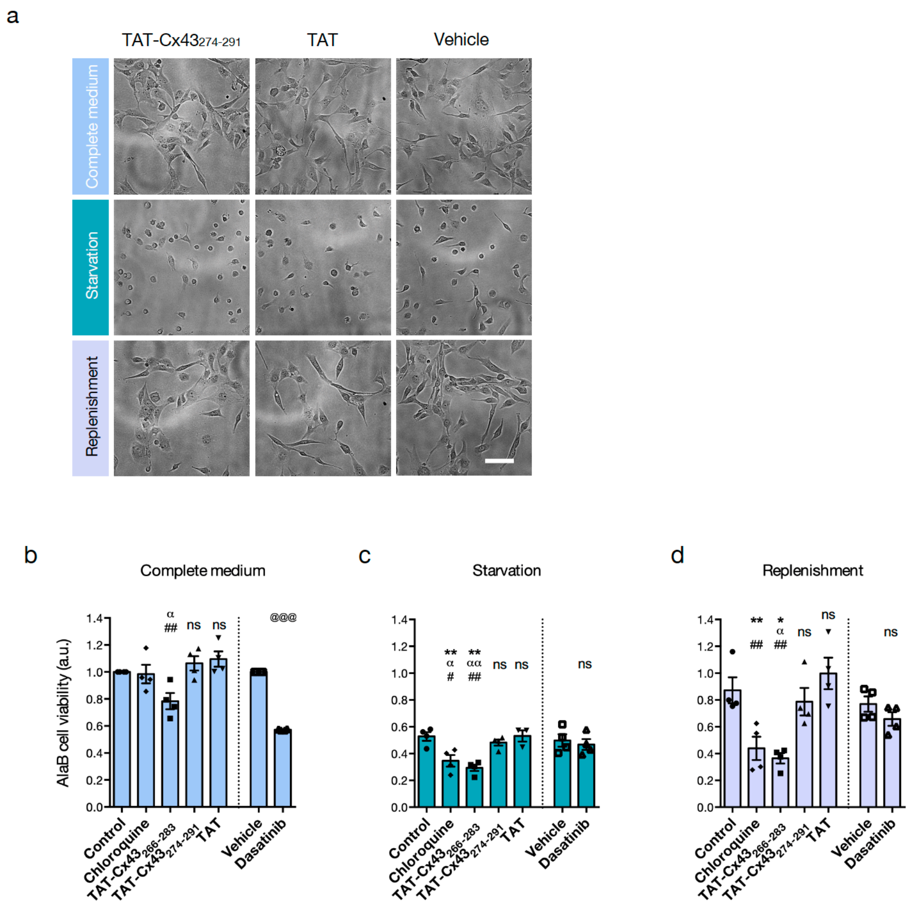

Figure S2. Viability of nutrient deprived dormant G166 GSCs. Related to Fig. 4. Treatment concentrations were the same as in Fig. 4 b-e: cell-penetrating peptides - 50 µM, dasatinib - 500 nM, CQ - 50 µM. (**a**) Representative cell culture fields imaged with an EVOS Floid imaging station at endpoint of the indicated conditions and treatments. Scale bar: 100 µm. (**b**) Alamar Blue assay was used to assess cell viability in the indicated conditions and treatments. All data are mean ± s.e.m. from at least 3 independent experiments. Each data point is the average of 2 technical replicates. $*P < 0.05$, $**P < 0.01$ vs control; $\alpha P < 0.05$, $\alpha\alpha P < 0.01$ vs TAT-Cx43$_{266-283}$; $\#P < 0.05$, $\#\#P < 0.01$ vs TAT; @@@P < 0.001 vs vehicle; ns, not significant.

Videos 1a-b. Related to Fig. 4. Nutrient starvation induced dormant G166 GSCs recorded by time-lapse microscopy as described in Materials and Methods and following the scheme in Fig. 4 a. (**a**) Control G166 GSCs were cultured in starvation medium for 24 h and then maintained in the same medium following the scheme in Fig. 4 a. Recording of starvation videos began after 24h in starvation medium and lasted for 48 h. (**b**) Then, the medium was replaced with complete medium without treatment and the cells were recorded for a further 48 h.

Videos 2a-b. Related to Fig. 4. Nutrient starvation induced dormant G166 GSCs treated with TAT-Cx43$_{266-283}$ recorded by time-lapse microscopy as described in Materials and Methods and following the scheme in Fig. 4 a. (**a**) G166 GSCs were cultured in starvation medium for 24 h and then 50µM TAT-Cx43$_{266-283}$ was added to the medium. Recording of starvation videos lasted for 48 h. (**b**) Then, the medium was replaced with complete medium without treatment and the cells were recorded for a further 48 h.

Videos 3a-b. Related to Fig. 4. Nutrient starvation induced dormant G179 GSCs recorded by time-lapse microscopy as described in Materials and Methods and following the scheme in Fig. 4 a. (**a**) Control G179 GSCs were cultured in starvation medium for 24 h and then maintained in the same medium following the scheme in Fig. 4 a. Recording of starvation videos began after 24h in starvation medium and lasted for 48 h. (**b**) Then, the medium was replaced with complete medium without treatment and the cells were recorded for a further 48 h.

Videos 4a-b. Related to Fig. 4. Nutrient starvation induced dormant G179 GSCs treated with TAT-Cx43$_{266-283}$ recorded by time-lapse microscopy as described in Materials and Methods and following the scheme in Fig. 4 a. (**a**) G179 GSCs were cultured in starvation medium for 24 h and then 50μM TAT-Cx43$_{266-283}$ was added to the medium. Recording of starvation videos lasted for 48 h. (**b**) Then, the medium was replaced with complete medium without treatment and the cells were recorded for a further 48 h.
